# Parturition among the Esquimaux

**Published:** 1900-03

**Authors:** 


### PARTURITION AMONG THE ESQUIMAUX.

The sexual life of the inhabitants of the farthest North is somewhat like that of animals. The long night is one of bodily torpor, the sexual functions are in abeyance and desire has failed. But with the return of the sun, the Esquimaux have a period of violent rut, and give themselves up to unbridled gratification of their pent-up lust. This carries out their resemblance to animals, particularly the seal, whose habits and the fierce fights of the sea-catch to get together and hold his sleek seraglio have been so well described by Kipling. The woman has to pay the piper, with ruinous interest, as may be gathered from the following description of parturition among the Esquimaux, which is taken from the *Medical Bulletin*, quoting the *Journal of the American Medical Association* :

Gleaves, in a recent article, has written of this subject, having had the opportunity of being in attendance during an Esquimau labor. He writes that it is seldom that a "cablona" (white man) doctor is permitted to witness this trying ordeal. It is the custom among Esquimaux not to be confined in *igloo*, or house. The woman must go into the woods or brush alone—no one being allowed to be present—and remain there with no food except a piece of dried fish for "five sleeps," when she is allowed to return to the *igloo*. Then she and her husband take a bath and change their undergarments. In the instance cited, Gleaves states that the birth of the child occurred in Alaska during January, when the temperature ranged from thirty to forty degrees below zero, and while the mother, when found, was secreted in the brush in a snow pit, oval in shape, about two feet deep and six across. The only warmth obtained was from a smoky fire made with twigs. She lay on a reindeer skin with no covering of any kind. "On my arrival at the parturition field I found the woman in labor on her knees with buttocks resting on her heels, and having severe bearing-down pains which came faster and faster and more severe until almost continuous, when the bag of waters ruptured. Hemorrhage occurred, but she soon rallied again and expelled the placenta, whereupon she took a piece of sinew which had been prepared from the hock of a caribou, and ligated the cord as close to the umbilicus as possible, then severed the cord close to the ligature with a piece of serrated flint. She washed the babe in the snow, although it rebelled by kicking and squalling at such a cold reception. The woman wore a belt or a piece of thong to confine her 'parka' around the waist, and to it was fastened, by short deerskin thongs, bits of ivory, buttons, leather bags in which she kept tobacco, matches and other small articles of value. After the snow-bath she placed her babe underneath the folds of her 'parka,' which is the usual place of the young Esquimau, and proceeded in a bent-over position with a staff in hand, stepping slowly and laboriously, leaving a trail of blood, to another snow pit about fifty feet away, which had been prepared. She would not remain any length of time at the place where the babe was born, for it is considered unclean." The mortality of both mothers and babes is said to compare favorably with our own death-rate.

